# Simultaneous sulfide and methane oxidation by an extremophile

**DOI:** 10.1038/s41467-023-38699-9

**Published:** 2023-05-23

**Authors:** Rob A. Schmitz, Stijn H. Peeters, Sepehr S. Mohammadi, Tom Berben, Timo van Erven, Carmen A. Iosif, Theo van Alen, Wouter Versantvoort, Mike S. M. Jetten, Huub J. M. Op den Camp, Arjan Pol

**Affiliations:** 1 Department of Microbiology, Radboud Institute for Biological and Environmental Sciences, Radboud University, Heyendaalseweg 135, 6525AJ Nijmegen, The Netherlands; 2 Present Address: Institute of Biogeochemistry and Pollutant Dynamics, Department of Environmental Systems Science, ETH Zurich, 8092 Zurich, Switzerland

**Keywords:** Bacterial physiology, Environmental microbiology, Metabolic pathways

## Abstract

Hydrogen sulfide (H_2_S) and methane (CH_4_) are produced in anoxic environments through sulfate reduction and organic matter decomposition. Both gases diffuse upwards into oxic zones where aerobic methanotrophs mitigate CH_4_ emissions by oxidizing this potent greenhouse gas. Although methanotrophs in myriad environments encounter toxic H_2_S, it is virtually unknown how they are affected. Here, through extensive chemostat culturing we show that a single microorganism can oxidize CH_4_ and H_2_S simultaneously at equally high rates. By oxidizing H_2_S to elemental sulfur, the thermoacidophilic methanotroph *Methylacidiphilum fumariolicum* SolV alleviates the inhibitory effects of H_2_S on methanotrophy. Strain SolV adapts to increasing H_2_S by expressing a sulfide-insensitive *ba*_3_-type terminal oxidase and grows as chemolithoautotroph using H_2_S as sole energy source. Genomic surveys revealed putative sulfide-oxidizing enzymes in numerous methanotrophs, suggesting that H_2_S oxidation is much more widespread in methanotrophs than previously assumed, enabling them to connect carbon and sulfur cycles in novel ways.

## Abstract (web summary)

Aerobic methanotrophic bacteria oxidize methane in sulfide-rich environments, even though hydrogen sulfide (H_2_S) inhibits methane oxidation and aerobic respiration. Here, Schmitz et al. show that a single microorganism can oxidize methane and H_2_S simultaneously, and this is associated with upregulation of a sulfide-insensitive terminal oxidase.

## Introduction

Hydrogen sulfide (H_2_S) is the most reduced form of sulfur (S) and a potent energy and sulfur source, toxicant, and signaling molecule^1–3^. It is a weak acid that easily diffuses through membranes and inhibits various processes such as aerobic respiration by binding to cytochrome *c* oxidases. In addition, other metabolic processes that use copper- and iron-containing enzymes are severely inhibited by H_2_S^1,4–6^. Hence, microorganisms living in sulfide-rich environments require adequate mechanisms to detoxify H_2_S^7,8^. In a myriad of environments, such as wetlands, marine sediments, soil, wastewater treatment plants, lakes, paddy fields, landfills, and acidic geothermal environments, H_2_S is produced through sulfate (SO_4_^2−^) reduction, mineralization of organic matter, and thermochemistry^8–18^.

Upon depletion of sulfate, organic matter is ultimately converted to methane (CH_4_) in oxygen-depleted ecosystems^9,12,13,19–21^. When both H_2_S and CH_4_ diffuse into the overlaying oxic zones, CH_4_ can be utilized as an energy source by aerobic methane-oxidizing bacteria, which are assumed to mitigate most emissions of this potent greenhouse gas^22^. Despite this effective methane biofilter, 548 to 736 Tg of CH_4_ is annually released into the atmosphere from various natural and anthropogenic sources^23,24^. Aerobic methanotrophs are part of various bacterial classes and families, including the ubiquitous Alpha- and Gammaproteobacteria^16,25,26^, Actinobacteria^27^ and the extremophilic *Methylacidiphilaceae* of the phylum Verrucomicrobia^28–31^. The latter are acidophilic bacteria that share a low pH optimum (2.0 − 3.5) and live between 35 and 60 °C^26,31,32^. All known verrucomicrobial methanotrophs have been isolated from geothermal habitats such as fumaroles and mudpots, from which large amounts of mostly thermogenic CH_4_ and H_2_S are emitted^16,28,33–35^. Geothermal environments are typically characterized by high H_2_S emissions and thus the verrucomicrobial methanotrophs isolated from these ecosystems are preeminent examples to study how methanotrophs are affected by H_2_S.

It is becoming increasingly clear that methanotrophs are metabolically versatile and able to use environmentally relevant energy sources such as H_2_, propane, ethane, acetate, acetone, 2-propanol, and acetol^16,36–38^. The ability to utilize various energy sources is highly beneficial in environments with heavily fluctuating gas emissions. Recently, it was demonstrated that pure cultures of the verrucomicrobial methanotroph *Methylacidiphilum fumariolicum* SolV can consume methanethiol (CH_3_SH), with the concomitant sub-stoichiometric formation of H_2_S, indicating that strain SolV partially metabolized toxic H_2_S^39^. Hereafter, an elegant study demonstrated that also proteobacterial methanotrophs can oxidize H_2_S^40^. The authors isolated the versatile alphaproteobacterium ‘*Methylovirgula thiovorans*’ strain HY1 from a South Korean peatland that could grow on thiosulfate (S_2_O_3_^2−^), tetrathionate (S_4_O_6_^2−^), elemental sulfur (S^0^), and a range of carbon compounds. However, strain HY1 cells grown on CH_4_ as sole energy source were not able to oxidize H_2_S, and H_2_S oxidation was only initiated and observed in cells grown in the presence of thiosulfate. In addition, growth on H_2_S was not studied. Considering recent observations, it is paramount to investigate whether microbes exist that can oxidize the environmentally relevant gases CH_4_ and H_2_S simultaneously, how methanotrophs cope with H_2_S and whether such methanotrophs can conserve energy and produce biomass using H_2_S as an energy source.

Here, through extensive chemostat cultivation, we show for the first time that a microorganism can oxidize CH_4_ and H_2_S simultaneously. *M. fumariolicum* SolV is inhibited by the presence of elevated H_2_S concentrations but H_2_S is rapidly oxidized to elemental sulfur (S^0^) as a detoxification mechanism to alleviate the inhibitory effect of H_2_S on CH_4_ oxidation. Strain SolV adapts to H_2_S with the upregulation of a Type III sulfide:quinone oxidoreductase (SQR) and an H_2_S-insensitive *ba*_3_-type cytochrome *c* oxidase, creating an electron transfer pathway from H_2_S to O_2_. Additionally, strain SolV incorporates ^13^CO_2_ using H_2_S as sole energy source. We propose that the H_2_S oxidation capacity of verrucomicrobial methanotrophs is essential to thrive in sulfur-rich acidic geothermal ecosystems. In addition, we found SQR in a plethora of proteobacterial methanotrophs of various environments. Considering CH_4_ and H_2_S coexist in a myriad of oxygen-limited ecosystems, H_2_S oxidation could be a trait present among many aerobic methanotrophs.

## Results

### Simultaneous H_2_S and CH_4_ oxidation, and chemolithoautotrophic growth on H_2_S

**Fig. 1:**
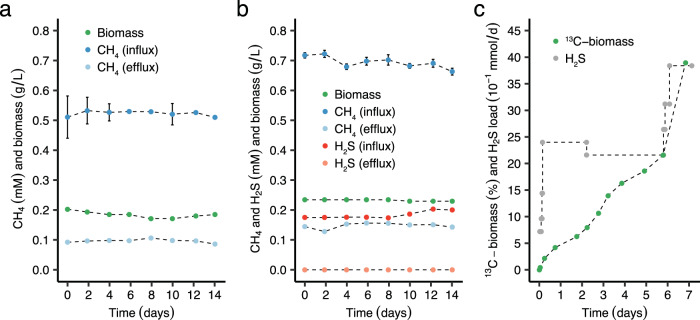
Growth of *M. fumariolicum* SolV at high loads of CH_4_ only, H_2_S and CH_4_, or H_2_S only. **a** Continuous culture oxidizing methane. **b** Continuous culture simultaneously oxidizing high concentrations of CH_4_ and H_2_S. **c** Fed-batch culture showing increase in ^13^C-biomass with H_2_S as sole energy source. Data are presented as mean ± standard deviations (*n* = 3 technical replicates). Source data are provided as a Source Data file.

The detection of genes encoding putative sulfide:quinone oxidoreductases (SQRs) in the genomes of verrucomicrobial methanotrophs prompted us to investigate whether methanotrophs can oxidize and adapt to H_2_S^16^. Accordingly, a continuous culture of the thermoacidophilic aerobic methanotroph *Methylacidiphilum fumariolicum* SolV (running as chemostat at a dilution rate (D) of 0.016 h^−1^) was maintained with CH_4_ as energy source and CO_2_ as carbon source (non-adapted cells; Fig. 1a), up to a load of 39 μmol CH_4_ min^−1^ · g DW^−1^ (Table 1). For comparison, a distinct continuous cultivation system was designed (with identical CH_4_ load) to adapt cells to increasing loads of H_2_S (Supplementary Fig. 1). The cells growing in this chemostat simultaneously oxidized H_2_S and CH_4_ (sulfide-adapted cells; Fig. 1b), up to concurrent loads of 42 μmol H_2_S · min^−1^ · g DW^−1^ and of 38 μmol CH_4_ · min^−1^ · g DW^−1^ (Table 1), while the H_2_S concentration in the gas outlet remained below 2 nmol · L^−1^. Steady state continuous cultures of non-adapted and sulfide-adapted cells could be maintained for many generations (Fig. 1a, b). Accumulation of elemental sulfur (S^0^) over weeks of growth was evident, as increasing amounts of a yellow precipitate (irregular microscopic particles) developed and attached to the metal parts and walls of the chemostat (Supplementary Fig. 2). After a few weeks of operation with H_2_S it was identified as being over 99% pure sulfur and the amount could account for at least 80% of the sulfide converted over this period. Through microscopy, only minute amounts of sulfur particles in the liquid could be observed as opposed to bacterial cells. Both non-adapted and sulfide-adapted cultures were operated under low dissolved O_2_ concentrations (1% air saturation) to minimize chemical sulfide oxidation. The low O_2_ concentrations also resulted in expression of hydrogenase activity as observed previously^41^. Control incubations in membrane-inlet mass spectrometry (MIMS) experiments without cells showed negligible oxidation of sulfide at micromolar range concentrations.

**Table 1:** Comparison of conversion and respiration rates of *M. fumariolicum* SolV cells from the CH_4_ chemostat (non-adapted cells) and the dual H_2_S-CH_4_ chemostat (sulfide-adapted cells)

	non-adapted cells	sulfide-adapted cells
Conversion rates in the chemostat ^a^		
CH_4_ conversion	39	38
H_2_S conversion	–	42
Max. H_2_S conversion (at <0.15 μM H_2_S and 1.7 μM O_2_)	–	156
Maximum conversion rates in the MIMS chamber ^b^		
CH_4_ conversion	200 ± 11	133 ± 9
H_2_ conversion	78 – 104	60 – 82
H_2_S conversion (at 5–30 μM H_2_S and <10 μM O_2_)	22 ± 4	120 ± 13
H_2_S conversion (at 5–30 μM H_2_S and 60–80 μM O_2_)	–	132 – 154
Maximum respiration rates in the MIMS chamber ^b^		
CH_4_ respiration ^c^	302 ± 9	211 ± 11
CH_3_OH respiration	311 ± 22	211 ± 13
H_2_ respiration	29 – 36	18 – 31
H_2_S respiration (at 40–80 μM H_2_S and <10 μM O_2_)	10 ± 1	53 ± 4
H_2_S respiration (at 30–80 μM H_2_S and 70–90 μM O_2_)	14 ± 1	77 ± 4

^a^Measured using GC and calculated from the differences between the gas inlet and gas outlet of the chemostat.

^b^Measured through membrane inlet mass-spectrometry (MIMS) and a fiber-optic oxygen sensor spot.

^c^This rate includes the theoretical 1 mol O_2_ needed to activate 1 mol CH_4_.

All rates are in μmol · min^−1^ · g DW^−1^. All CH_4_, CH_3_OH and H_2_ conversion and respiration rates measured in the MIMS chamber were determined in the absence of H_2_S. Respiration refers to O_2_ consumption in response to addition of the listed substrates.

Verrucomicrobial methanotrophs possess the Calvin-Benson-Bassham cycle for CO_2_ fixation^42^, raising the question whether they can grow as chemolithoautotroph on CO_2_ with H_2_S as energy source. Accordingly, a fed-batch reactor was inoculated with a diluted culture (OD_600_ = 0.05) of the dual H_2_S-CH_4_ chemostat and H_2_S and ^13^CO_2_ were supplemented as the only energy and carbon source, while the CH_4_ supply was disconnected. Over time, the biomass of *M. fumariolicum* SolV cells became enriched in carbon-13 by incorporating ^13^CO_2_ into biomass (Fig. 1c). When the H_2_S load was increased, the percentage of ^13^C-biomass increased accordingly. Growth was evident, as an increase in ^13^C-biomass was accompanied with an increase in dry weight (Supplementary Fig. 3). By quantifying H_2_S in the gas inlet and outlet of the reactor, H_2_S conversion efficiencies of ~98–100% were determined throughout the whole incubation period.

### H_2_S inhibition, oxidation, and adaptation to H_2_S

H_2_S consumption rates and inhibitory effects of H_2_S on *M. fumariolicum* SolV cells were measured inside a liquid-filled chamber connected to a membrane-inlet mass spectrometer (MIMS), which allows for the real-time and concurrent quantification of multiple gases, while O_2_ was measured by a sensor spot. A maximum CH_4_ conversion rate of non-adapted cells of 200 ± 11 μmol CH_4_ · min^−1^ · g DW^−1^ was measured with a concomitant O_2_ consumption rate of 302 ± 9 μmol O_2_ · min^−1^ · g DW^−1^ (Table 1). In comparison, for the sulfide-adapted cells a maximum CH_4_ conversion rate and concomitant O_2_ consumption rate of 33 and 30% lower was measured, respectively. Similarly, the maximum methanol respiration rates of sulfide-adapted cells were 32% lower than measured for the non-adapted cells (Table 1). Taking the 1 mol O_2_ required for the activation of 1 mol CH_4_ into account, the maximum CH_4_ respiration rates of the non-adapted and sulfide-adapted cells were about 3-fold lower compared to maximum methanol respiration rates (Table 1), indicating the conversion of methane to methanol as the rate limiting step. In addition, presumably due to the low dO_2_ concentration in the continuous cultures, the non-adapted and sulfide-adapted cells expressed a high hydrogenase activity (Table 1), with a measured H_2_:O_2_ consumption ratio of ~1:0.35 as expected^32,42^. As was the case for CH_4_ and methanol respiration, the maximum H_2_ respiration rates of the sulfide-adapted cells were lower than those of the non-adapted cells (Table 1). Hence, the gain in increased H_2_S oxidation capacity in sulfide-adapted cells comes at the expense of the CH_4_, methanol and H_2_ conversion capacities.

**Fig. 2:**
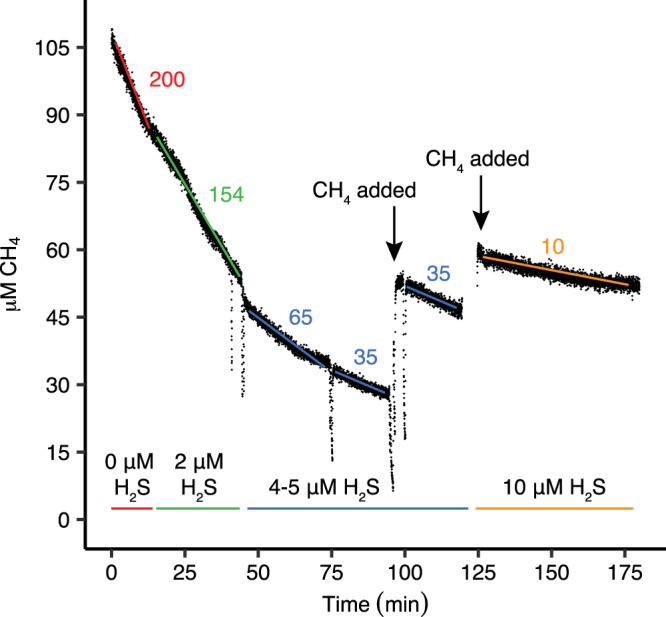
Inhibition of CH_4_ consumption by non-adapted *Methylacidiphilum fumariolicum* SolV cells in the presence of H_2_S. H_2_S was kept at various stable concentrations (indicated at the bottom) by pulse-wise additions of H_2_S to the MIMS chamber. Numbers indicate CH_4_ consumption rates in μmol CH_4_ · min^−1^ · g DW^−1^. At 170 min the MIMS chamber has become anoxic, resulting in cessation of CH_4_ consumption. Source data are provided as a Source Data file.

Sulfide-adapted cells in the chemostat oxidized H_2_S to low, non-inhibitory concentrations (Fig. 1b), which is necessary since the CH_4_ oxidation capacity of non-adapted cells (as well as sulfide-adapted cells) was affected by an H_2_S concentration as low as 1 μM. CH_4_ oxidation was inhibited by about 25%, 70–85% and 95% in the presence of 2 μM, 4-5 μM and 10 μM H_2_S, respectively (Fig. 2). Inhibition of CH_4_ conversion appeared reversible, as when H_2_S was consumed or flushed out of short-term incubations, CH_4_ conversion and CO_2_ production resumed immediately at their previous rates. After longer periods (2 h) of inhibition by 10–20 μM H_2_S, CH_4_ conversion rates were 25–35% lower. Whether these lower rates were the result of inhibition of pMMO or other parts of the respiratory chain as well could not be concluded as methanol (CH_3_OH) conversion was impaired as well after such long H_2_S exposures.

High initial O_2_ consumption rates were measured when only H_2_S was administered to non-adapted cells in the MIMS chamber. Interestingly, these rates immediately and rapidly decreased ~15-fold within a few minutes to stable rates of 10 ± 1 μmol O_2_ · min^−1^ · g DW^−1^ (at 40*–*80 μM H_2_S and <10 μM O_2_). This rapid decrease in respiration rate indicated the presence of sulfide-sensitive terminal oxidases (SSTOs) that were quickly inactivated after the addition of H_2_S and at least one type of sulfide-insensitive terminal oxidase (SITO) responsible for the remaining low respiration rate^43^. The maximum reaction rate of the SITO (10 ± 1 μmol O_2_ · min^−1^ · g DW^−1^) is limited, as it constitutes only 3% of the maximum respiration rate of these non-adapted cells on methanol (Table 1). At 10-fold higher O_2_ concentrations (70–90 µM O_2_ and 30*–*80 μM H_2_S), the remaining respiration rate increased ~40% (Table 1), suggesting that O_2_ is competing with H_2_S for the active site of the SSTOs, thereby alleviating H_2_S inhibition. SITO activity was cyanide sensitive as 95% of the respiration rate was inhibited at 1 mM potassium cyanide. The sulfide-adapted cells oxidized H_2_S with maximum O_2_ consumption rates of 53 ± 4 μmol O_2_ · min^−1^ · g DW^−1^ (Table 1). As at 40–80 μM H_2_S the SSTOs were assumed to be completely inhibited, these values represent the rates of the SITO, which are more than five times higher compared to the non-adapted cells (Table 1). H_2_S is primarily converted to elemental sulfur (S^0^), as a H_2_S:O_2_ stoichiometry of 1:0.48 ( ± 0.005; *n* = 3) was determined after simultaneous quantification of H_2_S and O_2_ consumption, together with the visible production of S^0^ (Supplementary Fig. 7b).

Maximum conversion rates of H_2_S at non-inhibiting, low (sub-micromolar) concentrations in the MIMS chamber were difficult to perform due to its rapid consumption that resulted in a variable inhibition. Alternatively, the maximum H_2_S conversion rates were determined in the dual H_2_S-CH_4_ chemostat by gradually increasing the sulfide load to 156 μmol H_2_S · min^−1^ · g DW^−1^ over the course of a day while monitoring the outlet concentration (Table 1). The latter increased from 2 to 25 nmol · L^−1^ and therefore remained below a liquid concentration of 40 nM, which was considered not to affect pMMO (as measured through MIMS incubations). Nevertheless, CH_4_ conversion did decrease about 40% but remained stable for days. When in a similar way the chemostat was given only H_2_S while CH_4_ was disconnected, the same maximum H_2_S conversion rate of 156 μmol H_2_S · min^−1^ · g DW^−1^ was measured. As respiration is not the limiting factor in this setup (Table 1), this rate is considered the maximum H_2_S conversion rate, which is 1.5 times higher than the SITO activity can account for in these sulfide-adapted cells and made possible by the SSTOs that were only partially inhibited at these low sulfide concentrations. Similar rates were measured for sulfide-adapted cells in the MIMS chamber in the presence of 60–80 μM O_2_ (Table 1). Hence, at low H_2_S and/or high O_2_ concentrations, the cells demonstrate the highest sulfide conversion rates, as the SSTOs are only partially inhibited. Noticeably, the above measured maximum H_2_S conversion rate in the MIMS chamber exceeded that of the maximum CH_4_ conversion rate of the sulfide-adapted cells (Table 1).

### Oxidation of methanol, H_2_ and formic acid in the presence of H_2_S

**Fig. 3:**
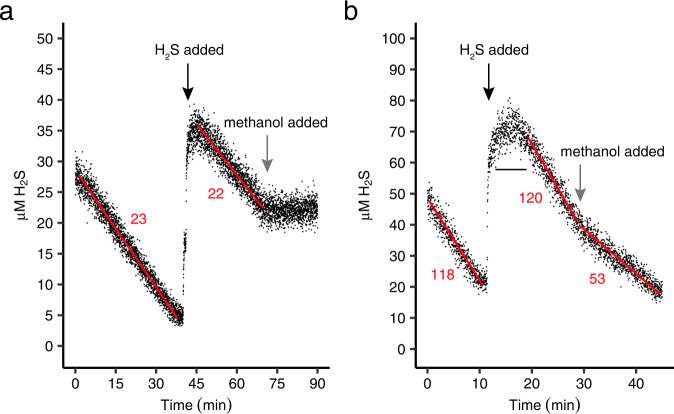
Inhibition of H_2_S consumption by *Methylacidiphilum fumariolicum* SolV cells in the presence of methanol. **a** Cessation of H_2_S consumption by non-adapted cells after the addition of methanol (final concentration 0.4 mM). **b** Inhibition of H_2_S consumption by sulfide-adapted cells after the addition of methanol (final concentration 5 mM). Numbers indicate consumption rates in μmol H_2_S · min^−1^ · g DW^−1^. The black horizontal line indicates a brief moment of anoxia to demonstrate H_2_S oxidation is dependent on O_2_. Source data are provided as a Source Data file.

Upon addition of methanol during respiration of 20–40 μM H_2_S by non-adapted cells in the MIMS chamber, H_2_S consumption ceased immediately (Fig. 3a) but the total respiration rate increased by ~40%. In contrast, H_2_S oxidation by sulfide-adapted cells (having five times higher SITO activity) continued at 43% of the rate when methanol was added (Fig. 3b), while the total respiration rate increased by ~25% (Supplementary Fig. 4). Hence, methanol and H_2_S were respired simultaneously and seem to compete for the same terminal oxidase. When sulfide (30 µM) was added to sulfide-adapted cells during methanol respiration, O_2_ consumption decreased ~3-fold (Supplementary Fig. 5). The remaining respiration rates (66 μmol O_2_ · min^−1^ · g DW^−1^) were higher than expected from the maximum (SITO-dependent) H_2_S respiration rate (53 ± 4 μmol O_2_ · min^−1^ · g DW^−1^), indicating that at least some methanol was still respired, which was confirmed by the fact that the CO_2_ production rate continued at 20–30% in the presence of sulfide. In contrast, at the same H_2_S concentrations, CH_4_ respiration had ceased almost completely (Fig. 2).

**Fig. 4:**
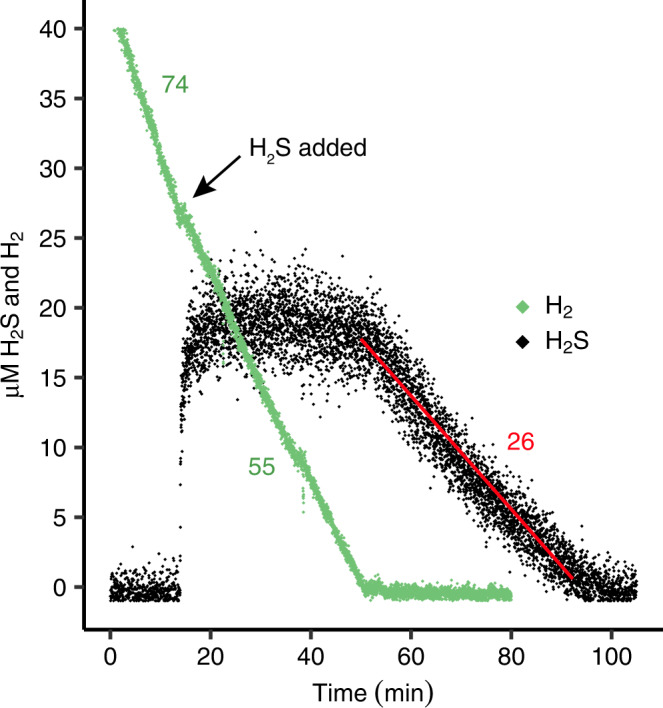
H_2_ and H_2_S consumption dynamics in non-adapted *Methylacidiphilum fumariolicum* SolV cells. Green numbers indicate H_2_ consumption rates in μmol · min^−1^ · g DW^−1^ before and after H_2_S addition, respectively. The red number and line indicate H_2_S consumption rate in μmol · min^−1^ · g DW^−1^ after depletion of H_2_. Source data are provided as a Source Data file.

Addition of H_2_S to non-adapted cells consuming H_2_ decreased the H_2_ consumption rate by 30%, while H_2_S consumption only started after complete depletion of H_2_ (Fig. 4). Furthermore, H_2_ respiration was up to two times higher than H_2_S respiration after all H_2_ had become depleted. Similarly, H_2_S oxidation at 20–40 μM H_2_S was reduced by about 80% upon addition of 200 μM formic acid (CHOOH) (Supplementary Fig. 6), while the total respiration rate increased by 15%. The observation that H_2_S is only oxidized after H_2_ (or methanol) has become depleted (Fig. 4) suggests competitive electron transfer pathways to the sulfide-insensitive terminal oxidase (SITO) due to its limited respiration capacity. Interestingly, when in a separate experiment 1.2 mM H_2_S was oxidized as sole energy source over ~3 h and O_2_ additions were stopped, H_2_S was produced under anoxic conditions (Supplementary Fig. 7). Conceivably, a sulfide-producing enzyme is being used by strain SolV, reducing the heretofore produced polysulfides and/or elemental sulfur (S^0^). In contrast, H_2_ was not consumed in the absence of polysulfides and/or elemental sulfur under anoxic conditions, indicating that these sulfur compounds and not sulfate present in the medium was used as electron acceptor. H_2_S production was stimulated up to 13 μmol H_2_S · min^−1^ · g DW^−1^ when H_2_ or methanol was present as electron donor.

### Kinetics of H_2_S oxidation and respiration

**Fig. 5:**
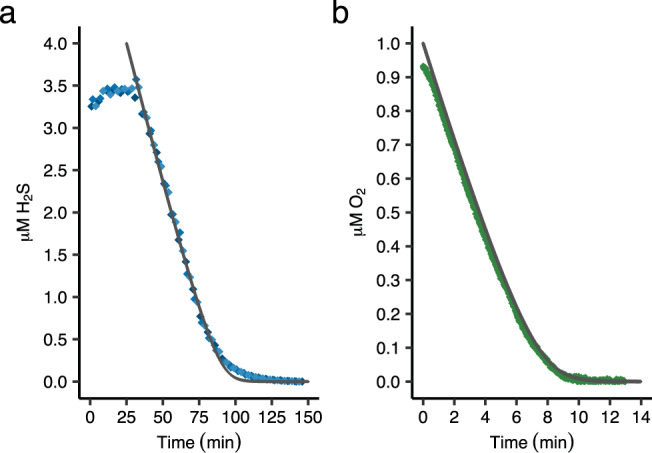
Kinetics of H_2_S oxidation by *Methylacidiphilum fumariolicum* SolV cells. **a** H_2_S oxidation measured through gas chromatography. Different blue shaded diamonds represent biological replicates (*n* = 3). The reaction was initiated by addition of cells after 33 min. **b** H_2_S respiration measured through a fiber-optic oxygen sensor spot in the MIMS chamber. Black lines indicate Michaelis-Menten curve fitting. The reaction was initiated by addition of cells at 0 min. Source data are provided as a Source Data file.

H_2_S oxidation kinetics by sulfide-adapted cells were studied using a gas chromatograph, which has a lower detection limit than the MIMS. Starting at 3.5 μM H_2_S and 190 μM O_2_, an almost linear decrease down to 1 μM H_2_S was observed with a rate of 167–223 μmol H_2_S · min^−1^ · g DW^−1^ (Fig. 5a). These rates are slightly higher than the maximum H_2_S conversion rates measured in the MIMS chamber at 5–30 μM H_2_S and 60–80 μM O_2_ (Table 1), which could be explained by the higher O_2_ and low H_2_S concentrations present in the incubations used for GC measurements. Because O_2_ and H_2_S compete for the sulfide-sensitive terminal oxidase (SSTO), a low H_2_S and high O_2_ concentration alleviate SSTO inhibition, leading to higher H_2_S consumption rates. Michaelis-Menten modeling of H_2_S consumption resulted in an apparent affinity constant *K*_s_ of 0.32 μM H_2_S. However, the H_2_S traces below 1 μM H_2_S did not follow the predicted curve and remained slightly above it (Fig. 5a). When in the MIMS chamber O_2_ consumption was followed down to zero at H_2_S concentrations of 15–20 μM an apparent affinity constant *K*_s_ of 0.14 ± 0.01 μM O_2_ was determined that follows Michaelis-Menten kinetics (Fig. 5b). In the presence of 15 μM H_2_S, SITO was not inhibited as identical O_2_ consumption rates were measured after sequential addition of O_2_ (Supplementary Fig. 8). Assuming only one terminal oxidase type to be active under these conditions and H_2_S conversion not being the limiting factor, a *K*_s_ of 0.14 ± 0.01 μM O_2_ could be a reliable value for the sulfide-insensitive terminal oxidase.

### Gene regulation in response to H_2_S

To assess how *M. fumariolicum* SolV cells adapt to H_2_S, mRNA from the dual H_2_S-CH_4_ chemostat (sulfide-adapted cells) and the CH_4_ chemostat (non-adapted cells), both in steady state, were extracted and gene expression was quantified (Table 2; Supplementary Fig. 9; Supplementary Data 1). In sulfide-adapted cells, the operon MFUM_v2_0219-21 was upregulated about 1.7-fold. The genes in this operon are annotated as NAD(FAD)-dependent dehydrogenase (MFUM_v2_0219), a protein homologous to the sulfur carrier protein TusA (MFUM_v2_0220) and a putative sulfur carrier protein DsrE2 (MFUM_v2_0221), respectively. A more detailed investigation revealed MFUM_v2_0219 to encode a type III sulfide:quinone oxidoreductase (SQR)^44^. Based on gene comparisons, a second gene (MFUM_v2_0138) might encode an SQR, although this gene was not significantly upregulated in the presence of H_2_S and transcribed to a much smaller degree than MFUM_v2_0219 in sulfide-adapted cells (Supplementary Data 1). Two genes (MFUM_v2_0873 and MFUM_v2_1149) were transcribed that might encode sulfur dioxygenases, which could putatively oxidize elemental sulfur to sulfite (SO_3_^2−^) (Supplementary Data 1). In addition, the genes MFUM_v2_0942 and MFUM_v2_0943 were upregulated 2-fold and 8-fold (Table 2) and show high similarity to genes encoding the cytochrome *c* protein SorB and sulfite:cytochrome *c* oxidoreductase SorA of *Thiobacillus novellus*, respectively^45^. In sulfide-adapted cells, the putative sulfur dioxygenase (MFUM_v2_0873) was transcribed to a similar degree as SQR (MFUM_v2_0219). However, based on the stoichiometry of 1 H_2_S: 0.48 O_2_ ( ± 0.005; *n* = 3) quantified in the MIMS chamber, the conversion of elemental sulfur and polysulfides via sulfite to sulfate is thought to have a minor role under the tested conditions. In addition, the oxidation of H_2_S was never accompanied by a decrease in pH, which would have been the case if elemental sulfur had been oxidized further to thiosulfate, sulfite or sulfate. The operon MFUM_v2_1257-61 encodes a *ba*_3_-type cytochrome *c* oxidase that was upregulated ~5-fold in the presence of H_2_S, agreeing with the 5-fold higher SITO respiration rate in sulfide-adapted cells. Interestingly, the highest upregulated gene (15-fold) encodes an 89 kDa heptahaem cytochrome *c* protein of unknown function (MFUM_v2_1950), showing highest similarity to genes found in thermophilic sulfide-oxidizers. In the presence of H_2_S, several genes encoding enzymes involved in the biosynthesis of sulfide for production of sulfur-containing metabolites (e.g., cysteine, methionine and glutathione) were heavily downregulated (Table 2). In addition, the downregulation of genes involved in CH_4_ oxidation and subsequent electron transfer in the respiratory chain was observed (Table 2). This downregulation is in accordance with the measured decreased maximum methane conversion and respiration rates.

**Table 2:** Regulation of genes of *M. fumariolicum* SolV cells grown in the dual CH_4_-H_2_S chemostat (sulfide-adapted cells) versus the CH_4_ chemostat (non-adapted cells)

ORF	Annotation	Upregulation factor
Genes involved in the oxidation of sulfur compounds and the respiratory chain		
MFUM_v2_0219	Sulfide:quinone oxidoreductase (sqr)	1.8
MFUM_v2_0220	Putative sulfur carrier protein	1.7
MFUM_v2_0221	Peroxiredoxin family protein	1.7
MFUM_v2_0942	Cytochrome c family protein	1.9
MFUM_v2_0943	Sulfite oxidase or related enzyme	8.3
MFUM_v2_1257	Conserved transmembrane protein of unknown function	4.5
MFUM_v2_1258	Conserved transmembrane protein of unknown function	5.4
MFUM_v2_1259	Cytochrome c oxidase (B(O/a)3-type) chain II (cbaB)	5.2
MFUM_v2_1260	Cytochrome c oxidase (B(O/a)3-type) chain I (cbaA)	4.5
MFUM_v2_1261	Conserved protein of unknown function	3.2
MFUM_v2_1950	Heptahaem-containing protein	15.7
MFUM_v2_1951	Putative starvation-inducible outer membrane lipoprotein	9.7

ORF	Annotation	Downregulation factor
Genes involved in assimilatory sulfide production		
MFUM_v2_0525	Sulfate adenylyltransferase subunit 1	11.6
MFUM_v2_0526	Sulfate adenylyltransferase subunit 2 (cysD)	18.7
MFUM_v2_0527	Phosphoadenosine phosphosulfate reductase (cysH)	29.4
MFUM_v2_0528	Homocitrate synthase 1 (nifV)	8.6
MFUM_v2_0573	Polysulfide reductase	2.2
MFUM_v2_0815	Sulfite reductase [NADPH] hemoprotein beta-component (cysI)	33.4
MFUM_v2_2220	O-acetylserine sulfhydrylase A (cysK)	3.0
Genes involved in methane oxidation		
MFUM_v2_1464	PqqA peptide cyclase PqqE	2.1
MFUM_v2_1604	Methane monooxygenase subunit alpha (pmoB3)	3.6
MFUM_v2_1605	Methane monooxygenase subunit beta (pmoA3)	3.0
MFUM_v2_1606	Methane monooxygenase subunit gamma (pmoC3)	2.9
MFUM_v2_1791	Methane monooxygenase subunit alpha (pmoB1)	1.6
MFUM_v2_1792	Methane monooxygenase subunit beta (pmoA1)	1.7
MFUM_v2_1793	Methane monooxygenase subunit gamma (pmoC1)	1.6
Genes involved in the respiratory chain		
MFUM_v2_2064	Succinate dehydrogenase flavoprotein subunit	1.6
MFUM_v2_2065	Succinate dehydrogenase cytochrome b subunit	2.5
MFUM_v2_2239	NADH-quinone oxidoreductase subunit D (nuoD)	1.6
MFUM_v2_2240	NADH-quinone oxidoreductase subunit C (nuoC)	1.6
MFUM_v2_2241	NADH-quinone oxidoreductase subunit B (nuoB)	1.6
MFUM_v2_2458	ATP synthase F1 complex subunit alpha (atpA)	1.7
MFUM_v2_2459	ATP synthase F1 complex subunit gamma (atpG)	1.6
MFUM_v2_2460	ATP synthase F1 complex subunit beta (atpD)	1.6
MFUM_v2_1602	Phosphoenolpyruvate synthetase (ppsA)	2.7

Listed genes have a basemean >4, an upregulation factor or downregulation factor >1.5 and an adjusted *p*-value ≤ 0.05 (all averages of triplicates). A two-sided Wald test was performed by DEseq2 to calculate adjusted *p*-values. *ORF* open reading frame.

### Phylogeny of putative SQRs in methanotrophs

The observation that *M. fumariolicum* SolV possesses an SQR and the fact that CH_4_ and H_2_S coexist in a large variety of environments prompted us to investigate the presence of SQR in methanotrophs. Indeed, genes encoding putative SQRs are also widespread in proteobacterial methanotrophs of various environments such as lakes, wetlands, rhizosphere, ocean sediments, permafrost soil, paddy fields, wastewater treatment plants, alkaline soda lakes, landfills and groundwater aquifers (Supplementary Fig. 10). SQRs are classified into six different types based on their structure, and differ in their affinity for H_2_S and their physiological role in the cell^44^. Putative SQRs were detected in a large variety of proteobacterial genera in which a pMMO and/or sMMO was present, such as *Crenothrix*, *Methylobacter*, *Methylocaldum*, *Methylocapsa*, *Methylococcus*, *Methylocystis*, *Methylohalobius*, *Methylomagnum*, *Methylomarinum*, *Methylomicrobium*, *Methylomonas*, *Methyloprofundus*, *Methylosinus*, *Methylospira*, *Methyloterricola*, *Methylotetracoccus*, *Methylotuvimicrobium* and *Methylovulum* (Supplementary Fig. 10). In addition, the recently isolated alphaproteobacterium ‘*Methylovirgula thiovorans*’ strain HY1 encodes a type I SQR^40^. In contrast, verrucomicrobial methanotrophs possess genes encoding a type III SQR, comprising bacterial and archaeal SQRs of which the least is known^44^.

## Discussion

In this study, we show for the first time that a microorganism can oxidize CH_4_ and H_2_S simultaneously, and that a methanotroph can produce biomass from CO_2_ with H_2_S as sole energy source. We showed that oxidation of H_2_S is necessary because H_2_S inhibits both respiration and CH_4_ oxidation. Cells responded to the presence of H_2_S by upregulating a type III sulfide:quinone oxidoreductase (SQR) and a sulfide-insensitive *ba*_3_-type terminal oxidase (SITO). In addition, we provide evidence for an H_2_S detoxification mechanism in methanotrophs, which, according to genomic information and the co-occurrence of methane and sulfide in a myriad of environments, seems to be widespread.

Very little is known about the effect of H_2_S on methanotrophy. A methanotrophic consortium sampled from a landfill showed decreased methanotrophic activity in the presence of H_2_S^46^. In addition, CH_4_ oxidation by *Methylocaldum* sp. SAD2, isolated from a sulfide-rich anaerobic digester, was significantly inhibited (44–60% decrease in methanol production) in the presence of 0.1% H_2_S, but the mechanism was not explored^47,48^. ‘*Methylovirgula thiovorans*’ strain HY1A was recently shown to be able to consume various reduced sulfur compounds together with CH_4_, but simultaneous oxidation of CH_4_ and H_2_S could not be observed^40^. In the peatland where strain HY1A was isolated from, the H_2_S concentration was below the detection limit, suggesting that a vigorous H_2_S detoxification might not be necessary. In contrast, the geothermal environments where *M. fumariolicum* SolV and other verrucomicrobial methanotrophs reside, are characterized by high concentrations of H_2_S (from <50 ppm to 20000 ppm)^28,35,49^. Accordingly, the demonstrated ability to fix CO_2_ with H_2_S as sole energy source and efficiently oxidize H_2_S to S^0^ could be highly advantageous in such harsh systems. Considering that in the natural environment multiple substrates coexist, a mixotrophic lifestyle, in which CH_4_, H_2_ and H_2_S are utilized simultaneously is expected to be more beneficial^32,50^.

H_2_S is known to bind to metals such as copper and iron, which could lead to inhibition of the CH_4_ oxidation capacity of the copper-dependent pMMO and terminal oxidases involved in the reduction of O_2_^1,4–6,51,52^. Interestingly, ‘*Methylovirgula thiovorans*’ strain HY1A only encodes an iron-dependent sMMO^40^, whereas *M. fumariolicum* SolV encodes three copper-dependent pMMOs^16^. The former strain does not simultaneously oxidize H_2_S and CH_4_, while the latter has a rapid H_2_S detoxification system to alleviate inhibition of methanotrophy. The extent to which a type of methane monooxygenase is inhibited by H_2_S could therefore influence the need for an H_2_S detoxification system. Because in *M. fumariolicum* SolV the gene encoding a type III SQR was upregulated in the presence of H_2_S, we propose that this enzyme is responsible for the observed oxidation of H_2_S to elemental sulfur. Indeed, type III SQRs were shown to couple the oxidation of H_2_S to the reduction of quinones in several archaea and bacteria^53,54^. In verrucomicrobial methanotrophs, three different types of terminal oxidases are found: an *aa*_3_-type, a *ba*_3_-type, and a *cbb*_3_-type^16^. Possessing multiple types of terminal oxidases allows a branched electron transport chain, which is highly advantageous in environments with fluctuating conditions and varying substrate and oxygen availability. Through respiration studies, we showed that *M. fumariolicum* SolV possesses one or more sulfide-sensitive terminal oxidases (SSTO) and at least one sulfide-insensitive terminal oxidase (SITO). Because a *ba*_3_*-*type terminal oxidase is strongly upregulated in cells growing at high H_2_S loads, we propose this specific enzyme complex to be the dedicated SITO in verrucomicrobial methanotrophs. Similarly, in sulfur-grown cells of *Acidithiobacillus ferrooxidans* this *ba*_3_-type oxidase was upregulated^55^. The highly upregulated heptahaem cytochrome *c* protein (MFUM_v2_1950) in *M. fumariolicum* SolV might be involved as electron carrier from SQR to the electron transport chain. This putative electron carrier could explain why H_2_S respiration still partially continues upon addition of methanol in the sulfide-adapted cells and not in the non-adapted cells. In the latter, the lack of this putative heptahaem electron carrier could be the limiting factor for H_2_S respiration, being overruled by the relatively large amounts of the electron carrier XoxGJ, mediating electron transfer from methanol to the terminal oxidase^56^. In contrast, in non-adapted cells the ratio in transcripts of the genes encoding XoxGJ and the putative heptahaem electron carrier is 27.6 compared to 1.2 in sulfide-adapted cells. Accordingly, the upregulation of the gene encoding the heptahaem electron carrier might enable sulfide respiration to occur concurrently with methanol oxidation, using the same terminal oxidase. H_2_S impedes both the SSTO, and the reaction catalysed by pMMO, as at 10 μM H_2_S the conversion of CH_4_ was almost completely inactivated while methanol, formate and H_2_ conversion could still proceed. The observed decrease in CH_4_ conversion in the chemostat at a maximum H_2_S load of 156 μmol H_2_S · min^−1^ · g DW^−1^ (liquid concentration <40 nM) was more than can be expected from our methane conversion inhibition studies and may indicate that a large portion of the respiratory chain is used for electrons generated by H_2_S oxidation, resulting in an overreduced Q-pool which prohibits proper functioning of alternative complex III (ACIII). Oxidation of H_2_S is needed to keep this molecule at low, non-inhibitory concentrations. Consequently, the electrons released from this oxidation need to be processed by the electron transport chain, leading to substrate competition during the simultaneous oxidation of multiple compounds such as H_2_S and CH_4_. Similarly, it was proposed that an overactive SQR in *Rhodobacter capsulatus* could lead to an overreduction of the quinone pool^57^. The upregulated *ba*_3_*-*type oxidase may alleviate this problem by oxidizing quinol and reducing the terminal electron acceptor O_2_. In *Aquifex aeolicus*, a related *ba*_3_-type oxidase was found in a supercomplex with SQR^58^. This terminal oxidase was shown to not only oxidize reduced cytochrome *c*, but also ubiquinol directly^59^. In strain SolV there may be an important role for the highly upregulated heptahaem protein as a dedicated electron shuttle between the quinone-accepting ACIII and the *ba*_3_-type oxidase. A branched electron transport chain with different terminal oxidases enables metabolic versatility and adaptations. For example, *E. coli* uses the proton-pumping *bo*_3_-type oxidase during growth but requires the sulfide-insensitive *bd*-type oxidases to keep growing in the presence of H_2_S^60^. Interestingly, two genes are present that could encode sulfur dioxygenases (MFUM_v2_0873 and MFUM_v2_1149) to further oxidize elemental sulfur. However, the measured stoichiometry of 1 H_2_S to 0.48 O_2_, the production of elemental sulfur and absence of acid production clearly show that H_2_S is not oxidized further to a significant extent. It remains to be investigated if methanotrophs can oxidize H_2_S further to sulfite and sulfate.

Cells of *M. fumariolicum* SolV were shown to rapidly oxidize H_2_S with a low apparent affinity constant (*i.e*., high affinity) below 1 μM H_2_S. The observed kinetic values are not surprising, since H_2_S already inhibits methanotrophy at such low concentrations. Through gas chromatography, an exact apparent affinity constant for whole cells could not be determined, as H_2_S consumption did not follow a typical Michaelis-Menten curve. A limitation of the respiratory capacity for H_2_S oxidation above about 1 μM may explain such deviation and could be resolved by purification of SQR. The observation that *M. fumariolicum* SolV reduced elemental sulfur or polysulfides to H_2_S in the presence of H_2_ or methanol is intriguing. ‘*Methylovirgula thiovorans*’ strain HY1A grown on thiosulfate increasingly produced an enzyme that resembles a protein known to possess sulfhydrogenase activity^40^. Interestingly, this enzyme clusters with the group 3b [NiFe] hydrogenase of *M. fumariolicum* SolV, thought to be involved in the production of NAD(P)H for CO_2_ fixation^50^. Indeed, it is proposed that these hydrogenases can have sulfhydrogenase activity, which could be a mechanism to dispose of reducing equivalents^61,62^. Hence, the group 3b [NiFe] hydrogenase of *M. fumariolicum* SolV might be responsible for the conversion of S^0^ to H_2_S. Culturing in chemostats again turned out to be a very powerful tool to investigate the metabolism of methanotrophs^41,63^. Through adaption, *M. fumariolicum* SolV was able to respire H_2_S at a rate five times higher than non-adapted cells, presumably due to the upregulation of SQR and the *ba*_3_-type terminal oxidase.

Verrucomicrobial methanotrophs that thrive in geothermal environments possess a clear mechanism to cope with H_2_S. Accordingly, SQR and a sulfide-insensitive terminal oxidase could enable these methanotrophs to thrive in H_2_S-rich environments. Indeed, pyrosequencing showed that *Methylacidimicrobium*-related 16 S rRNA gene sequences were abundantly present in the crown of concrete sewage pipes rich in CH_4_ and H_2_S^64^. Concerning proteobacterial methanotrophs, the effect of H_2_S warrants further investigation. Because aerobic methanotrophs live in environments in which H_2_S is often present, we propose that the mechanism of H_2_S detoxification is widespread in methanotrophs in various environments.

## Methods

### Microorganism and culturing

*Methylacidiphilum fumariolicum* SolV used in this study was isolated from a mud pot of the Solfatara near Naples, Italy^28^. The genome of this strain is publicly available and accessible at Genoscope [https://mage.genoscope.cns.fr/microscope/mage/viewer.php?O_id=1176], as well as at EMBL/NCBI (BioProject PRJEA85607; accession ERS14853105). This environment is characterized by large sulfide emissions, high temperatures and extremely low pH values. The growth medium was composed of 0.2 mM MgCl_2_, 0.2 mM CaCl_2_, 1 mM Na_2_SO_4_, 2 mM K_2_SO_4_, 7.5 mM (NH_4_)_2_SO_4_ and 1 mM NaH_2_PO_4_ and trace elements at final concentrations of 1 μM NiCl_2_, 1 μM CoCl_2_, 1 μM MoO_4_Na_2_, 1 μM ZnSO_4_, 1 μM CeCl_3_, 5 μM MnCl_2_, 5 μM FeSO_4_, 10 μM CuSO_4_ and 40–50 μM nitrilotriacetic acid (NTA). Cells were grown as methane-limited continuous culture at 55 °C as described before^65^, except that the pH was regulated at 2.5–3.0, that a small 400 mL chemostat was used with medium as described above and that H_2_ was not supplemented. The oxygen concentration was regulated at 1% air saturation. In addition, a second chemostat was operated under similar conditions, but to which H_2_S was added through an additional gas inlet (Supplementary Fig. 1). H_2_S was produced by mixing 100 mM anoxic Na_2_S and 210 mM HCl in a 50 mL bottle with a peristaltic pump. The argon/CO_2_ (95%/5%, v/v) gas stream to the reactor was led through this bottle. In order to determine the maximum H_2_S conversion rate of the chemostat, the cells were gradually exposed to higher H_2_S concentrations by regulating the peristaltic pump. The H_2_S concentrations in the gas inlet and gas outlet were determined using gas chromatography (described in the subsection: Batch incubations and gas chromatography). Because H_2_S was supplied through the gas inlet and therefore needs to be transferred to the liquid phase, the liquid H_2_S concentration will be close to or lower than its equilibrium concentration, which at 55 °C is 1.6 times the gas concentration (calculated from the Ostwald coefficient at 55 °C)^66^. In addition, to observe whether *M. fumariolicum* SolV can grow on H_2_S as sole energy source a fed-batch culture was operated in the same setup as the chemostat system. In this case the medium flow was stopped, and the argon/CO_2_ gas was changed for an argon only gas stream. At the same time equal amounts of a ^13^C-labeled bicarbonate solution (50 mM) and HCl solution (100 mM) were additionally added to the sulfide mixing bottle, creating a ^13^C-CO_2_ gas concentration of about 2%. 5 mL biomass samples from the fed-batch culture were collected by centrifugation over several days and the pellets were washed with acidified water (pH 3). Pellets were then resuspended in small amounts of acidified water and samples were subsequently pipetted into tin cups and dried overnight at 70 °C under vacuum. ^13^CO_2_ incorporation into biomass was assessed by measuring the ^13/12^C ratio using a Finnigan DeltaPlus isotope-ratio mass spectrometer (IR-MS) as described before^42^.

### Membrane-inlet mass spectrometry and respiration measurements

To accurately measure dissolved gases, membrane-inlet mass spectrometry (MIMS) was performed as described previously^65^, except that a 30 mL MIMS chamber was used. All rates were measured at 52 °C. The inserted probe consisted of a blunt end stainless steel tube (diameter 3 mm) that was perforated with 4–16 holes of 1 mm diameter. The holes were covered with silicon tubing (Silastic, 50VMQ Q7-4750 Dow Corning, supplied by Freudenberg Medical via VWR international, 1.96 mm outer diameter x 1.47 mm inner diameter). For easy mounting the silicon tubing was soaked briefly in hexane, which causes silicone to swell. The metal part was wetted with iso-propanol as lubricant. The probe was directly connected via a 1/8- or 1/16-inch stainless steel tube to the MS that was operated at 40 μA emission current. Medium with a pH equal to that of the culture (pH 2.5–3.0) added to the chamber was first flushed with 3% CO_2_ in argon gas after which the oxygen concentration was adjusted to the desired value by adding pure oxygen gas or air via the headspace. Mass 15 and 16 are both dominant masses for CH_4_ in the mass spectrometer, but mass 15 has a much lower background signal than mass 16 and was therefore chosen to measure CH_4_. Methane and hydrogen (mass 2) were added as a gas in the headspace or, in the case of calibration, from a saturated stock solution. These stock solutions were prepared in a closed bottle with water at room temperature and a headspace of pure gas with known pressure. For the solubility in water 1.47 mM and 0.80 mM were taken for methane and hydrogen, respectively (at 22 °C and 1 bar)^66^. When CO_2_ production rates were to be measured, ^13^C-bicarbonate and equimolar amounts of sulfuric acid were added after flushing with argon. In this way the simultaneously occurring CO_2_ fixation is mainly from ^13^C-labeled CO_2_ (mass 45), leading to less interference with measurement of CO_2_ production. At the start, unlabeled CO_2_ (mass 44) was very low, and its increase reflected almost exclusively CO_2_ production from unlabeled methane or methanol.

The stoichiometry of H_2_S oxidation was determined through pulse-wise additions of a sulfide stock solution and O_2_ (as tiny gas bubbles with a syringe) in order to keep concentrations low at 1–20 μM H_2_S and 0–5 μM O_2_. In total, 0.7–1.4 mM of Na_2_S was added over a period of 1.5–3 h. During this experiment, equimolar amounts of a 200 mM sulfuric acid stock solution were added simultaneously to limit the pH change within 0.2 units. The oxygen concentration was simultaneously measured in the MIMS chamber by means of a fiber-optic oxygen sensor spot (TROXSP5, PyroScience, Aachen, Germany) that was glued on the inside of the chamber. These spots could measure down to about 20 nM oxygen, which is much lower than can be measured with the mass 32 signal of MIMS.

### Batch incubations and gas chromatography

To determine kinetic parameters of H_2_S oxidation by sulfide-adapted cells, batch incubations were performed in 120 mL serum bottles containing 10 mL medium without any trace elements. Trace elements were omitted to minimize the effect of abiotic sulfide oxidation. The bottles were closed with butyl rubber stoppers. Incubations were performed at 55 °C and 350 rpm. H_2_S was prepared by mixing Na_2_S with HCl in a closed bottle. A volume headspace was taken and injected into 120 mL serum bottles and equilibrated for 30 min before initiating the assay by addition of cells. H_2_S was measured by injecting 100 μL of the headspace of the bottles with a Hamilton glass syringe into a GC (7890B GC systems Agilent technologies, Santa Clara, USA) equipped with a Carbopack BHT100 glass column (2 m, ID 2 mm) and a flame photometric detector (FPD). The areas obtained were used to calculate H_2_S amounts using calibration standard curves with H_2_S. Briefly, 400 μL of a 25 mM Na_2_S stock (sodium sulfide nonahydrate, purity >98%, Sigma-Aldrich) was acidified with 2 mL 0.5 M HCl in a 574 mL bottle creating a headspace concentration of 17.4 nmol · mL^−1^. Small volumes of the headspace were subsequently added to a 1162 mL bottle to create various H_2_S concentrations to be injected (0.1 mL) into the GC for calibration. The calibration curve ranged from ~1 nmol · L^−1^ to 1 μmol · L^−1^ H_2_S.

### RNA isolation, transcriptomics, and data analysis

For each replicate, 10 mL was sampled from the chemostat, and cells were immediately pelleted for 3 min at 15,000 × *g*, snap-frozen in liquid nitrogen and stored at –80 °C. Cells were harvested from cultures in steady state, which corresponds to constant parameters over at least 5 reactor volume changes. Total RNA was isolated using the RiboPure™ RNA Purification Kit for bacteria (Thermo Fisher Scientific, Waltham, MA, USA) according to the manufacturer’s protocol. Ribosomal RNA was removed from the total RNA samples to enrich for mRNA using the MICROBExpress™ Bacterial mRNA Enrichment Kit (Thermo Fisher Scientific) according to the manufacturer’s protocol. The Qubit™ RNA HS Assay Kit (Thermo Fisher Scientific) and the Agilent RNA 6000 Nano Kit (Agilent Technologies, Waldbronn, Germany) and protocols were used for the quantitative and qualitative analysis of the extracted total RNA and enriched mRNA. The latter was used for library preparation by using the TruSeq Stranded mRNA Reference Guide (Illumina, San Diego, CA, USA) according to the manufacturer’s protocol. For quantitative and qualitative assessment of the synthesized cDNA, the Qubit™ dsDNA HS Kit (Thermo Fisher Scientific) and the Agilent High Sensitivity DNA kit (Agilent Technologies) and protocols were used. Transcriptome reads were checked for quality using FastQC^67^ and subsequently trimmed 10 base pairs at the 5' end and 5 base pairs at the 3' end of each read. Reads were mapped against the *M. fumariolicum* SolV complete genome (accession number LM997411)^68^ using Bowtie2^69^. The remainder of the analysis and the production of images was performed in version 4.0.2 of the R environment^70^. The mapped read counts per gene were determined using Rsubread^71^ and fold change and dispersion were estimated using DEseq2^72^. Before doing any statistics, principal component analysis on the top 1000 genes by variance of each sample was performed to check whether samples within the same condition were both similar to each sample part of the same condition, and dissimilar to any other sample. For differential expression, a Wald test was employed by DEseq2 to calculate adjusted *p*-values. Differences in counts were considered to be significant if the basemean was >4, the log_2_-fold change was higher than [0.58] and the adjusted *p*-value was ≤0.05. For easy comparisons between samples, TPM (Transcripts Per Kilobase Million) values were calculated.

### TOC measurements

The total organic carbon (TOC) concentrations of the cultures were determined using a TOC-L CPH/CPN analyzer (Shimadzu, Duisburg, Germany). Samples were diluted three times in Milli-Q water before measurements and subsequently sparged for 20 min with ozone while stirring to remove CO_2_ from the liquid. Acidification of the solutions was not needed due to the low pH of the samples. An optical density of 1 measured at 600 nm is equivalent to ~450 mg dry weight (DW) per litre.

### Phylogenetic analysis

All available genome sequences of known methylotrophs from the orders Methylococcales (Gammaproteobacteria) and Methylacidiphilales (Verrucomicrobia), the families *Methylocystaceae* and *Beijerinckia* (Alphaproteobacteria), and the genus *Methylomirabilis* were retrieved from the NCBI database. Genomes of methanotrophs were selected by blasting amino acid sequences of PmoA from *Methylococcus capsulatus* (SwissProt accession Q607G3) and sMMO from *Methylosinus acidophilus* (NCBI accession AAY83388.1) with an e-value threshold of 10^−3^ and a %-id threshold of >30%. Genomes containing a methane monooxygenase sequence were subsequently mined for putative SQR sequences by blasting a representative sequence of each of the SQR subtypes as defined by previous research^44^: type I, WP_010961392.1; type II, WP_011001489.1; type III, WP_009059890.1; type IV, WP_011372252.1; type V, WP_012502121.1; type VI, WP_011439951.1. Putative SQR sequences were aligned with those in the phylogenetic tree of^44^ using Muscle 3.8.1551^73^ with default settings. A maximum-likelihood phylogenetic tree with 500 bootstrap replicates was constructed using RAxML 8.2.10^74^ using the rapid bootstrapping option and the LG amino acid substitution model^75^. The final tree was visualized using MEGA7 and the clade of flavocytochrome *c* sulfide dehydrogenase (FCSD) sequences was used as outgroup.

### Reporting summary

Further information on research design is available in the Nature Portfolio Reporting Summary linked to this article.

## Supplementary information

Supplementary Information

Peer Review File

Description of Additional Supplementary Files

Supplementary Dataset 1

Reporting Summary

## Data Availability

The RNA sequencing data in this study have been deposited in the NCBI database under accession number PRJNA766544. The genome of *Methylacidiphilum fumariolicum* SolV has been deposited in the NCBI database under accession number ERS14853105. Supplementary Data 1 and the Source Data file are also available on figshare (10.6084/m9.figshare.22779005). Source data are provided with this paper.

## Source data

Source Data
