# Supplementary material for: Simultaneous sulfide and methane oxidation by an extremophile
Source: Nat Commun. 2023 May 23;14:2974. doi: 10.1038/s41467-023-38699-9 (PMC10205796; doi:10.1038/s41467-023-38699-9)
Supplement: Supplementary file 1 — Supplementary Information [file 41467_2023_38699_MOESM1_ESM.pdf]

SUPPLEMENTARY INFORMATION

Simultaneous sulfide and methane oxidation by an extremophile

Rob A. Schmitz[1,2], Stijn H. Peeters[1], Sepehr S. Mohammadi[1], Tom Berben[1], Timo van Erven[1], Carmen A. Iosif[1], Theo van Alen[1], Wouter Versantvoort[1], Mike S.M. Jetten[1], Huub J.M. Op den Camp[1*] & Arjan Pol[1]

[1] Department of Microbiology, Radboud Institute for Biological and Environmental Sciences, Radboud University, Heyendaalseweg 135, 6525AJ, Nijmegen, The Netherlands.

[2] Present address: Institute of Biogeochemistry and Pollutant Dynamics, Department of Environmental Systems Science, ETH Zurich, 8092 Zurich, Switzerland.

*Correspondence: Prof. dr. Huub J. M. Op den Camp (h.opdencamp@science.ru.nl)

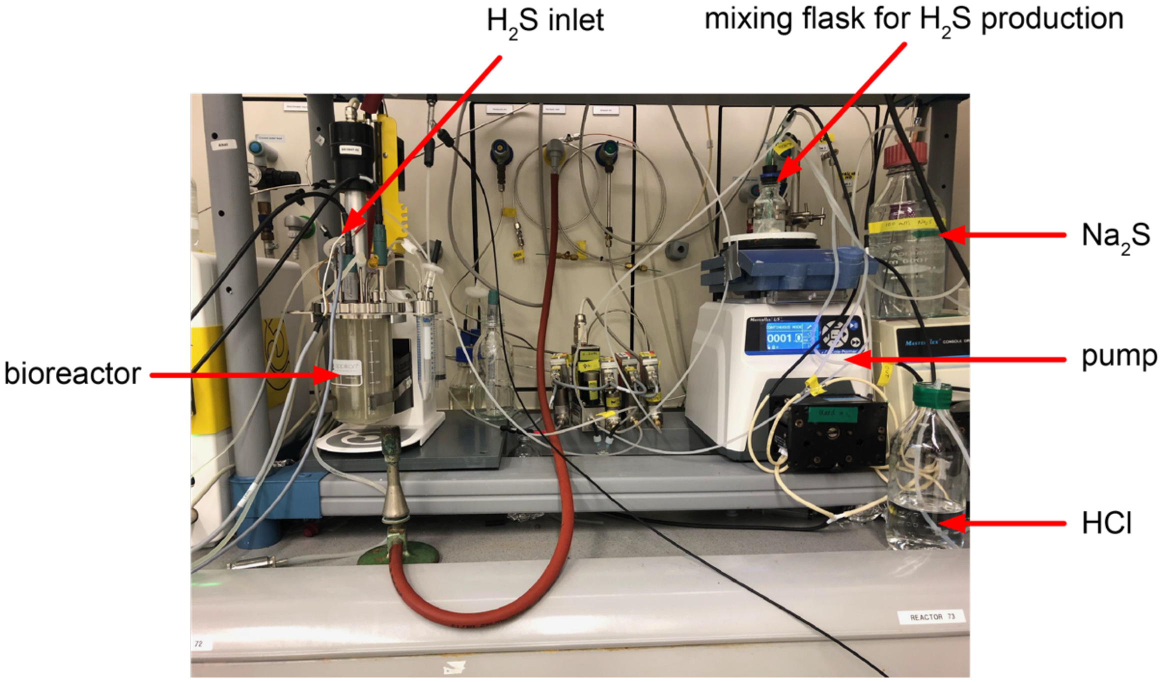

Supplementary Figure 1. System to continuously culture *Methylacidiphilum fumariolicum* SolV cells on H_2S and CH_4. H_2S is produced externally in a mixing flask by mixing sodium sulfide (Na_2S) and hydrochloric acid (HCl) and is subsequently fed into the chemostat through a gas inlet. The entire system was operated inside a fumehood.

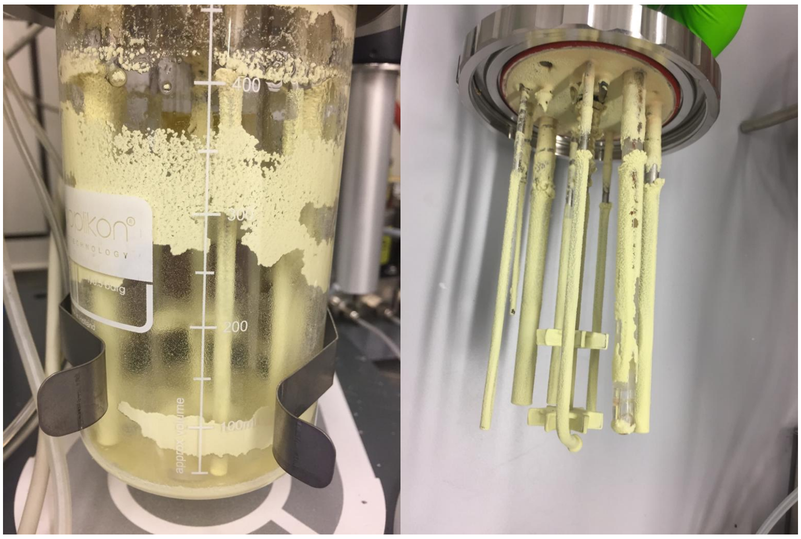

Supplementary Figure 2. Yellow deposits of elemental sulfur from the oxidation of H_2S by *Methylacidiphilum fumariolicum* SolV after growing for weeks in a continuous culture under high loads of H_2S and CH_4.

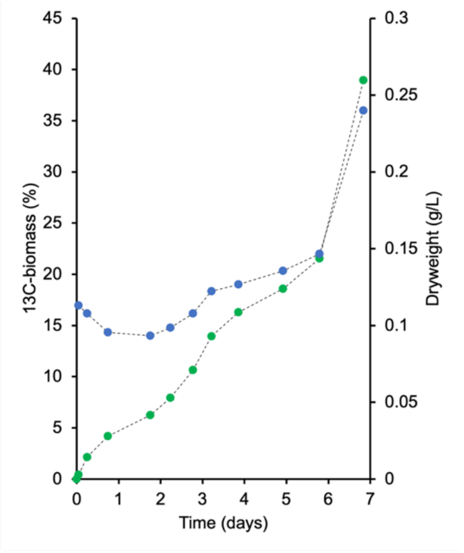

Supplementary Figure 3. Increase in ^{13}C-biomass (green datapoints) and dryweight (blue datapoints) of *M. fumariolicum* SolV cells grown in a fed-batch bioreactor with $^{13}CO_2$ as carbon source and H_2S as sole energy source. Source data are provided as a Source Data file.

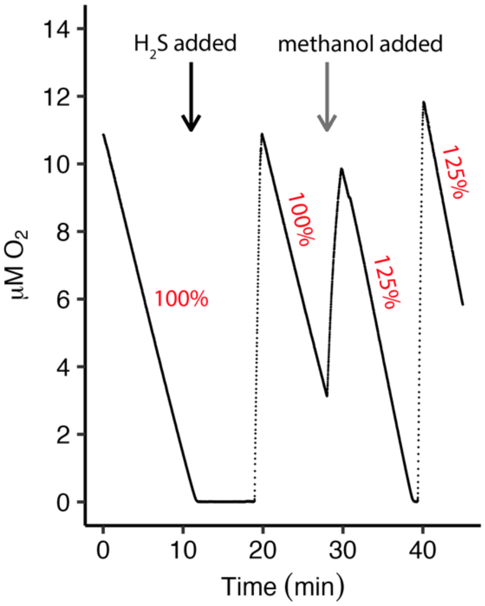

Supplementary Figure 4: Effect of the addition of methanol (5 mM) on respiration by sulfide-adapted *Methylacidiphilum fumariolicum* SolV cells. Percentages indicate respiration rates, in which the H_2S respiration rate is set at 100%. The respiration rates accompany the H_2S consumption rates in Figure 3b during the same timeframe. Each peak represents the timepoint at which O_2 was added. O_2 was measured using a fiber-optic oxygen sensor spot in the MIMS chamber. Source data are provided as a Source Data file.

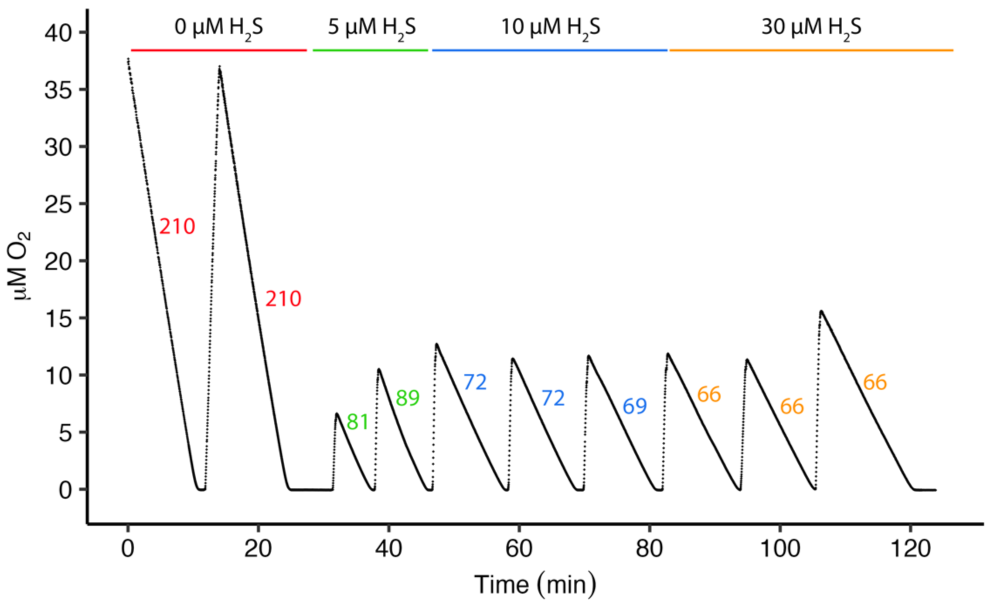

Supplementary Figure 5. Inhibition of methanol (10 mM) respiration by sulfide-adapted *Methylacidiphilum fumariolicum* SolV cells in the presence of H_2S. Numbers indicate respiration rates in µmol $O_2 \cdot min^{-1} \cdot g\ DW^{-1}$. H_2S was kept at various stable concentrations by pulse-wise additions of H_2S to the MIMS chamber. Each peak represents the timepoint at which O_2 was added. Source data are provided as a Source Data file.

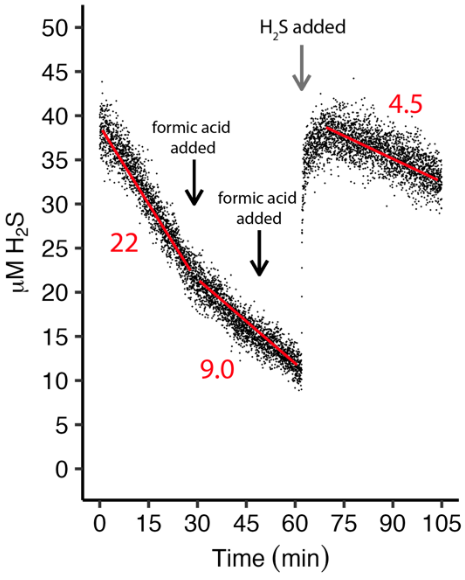

Supplementary Figure 6. Inhibition of H_2S consumption by non-adapted *Methylacidiphilum fumariolicum* SolV cells in the presence of formic acid. Formic acid was added twice to create a final concentration of 200 µM. Numbers indicate consumption rates in µmol $H_2S \cdot min^{-1} \cdot g\ DW^{-1}$. Rates were measured in the MIMS chamber. Source data are provided as a Source Data file.

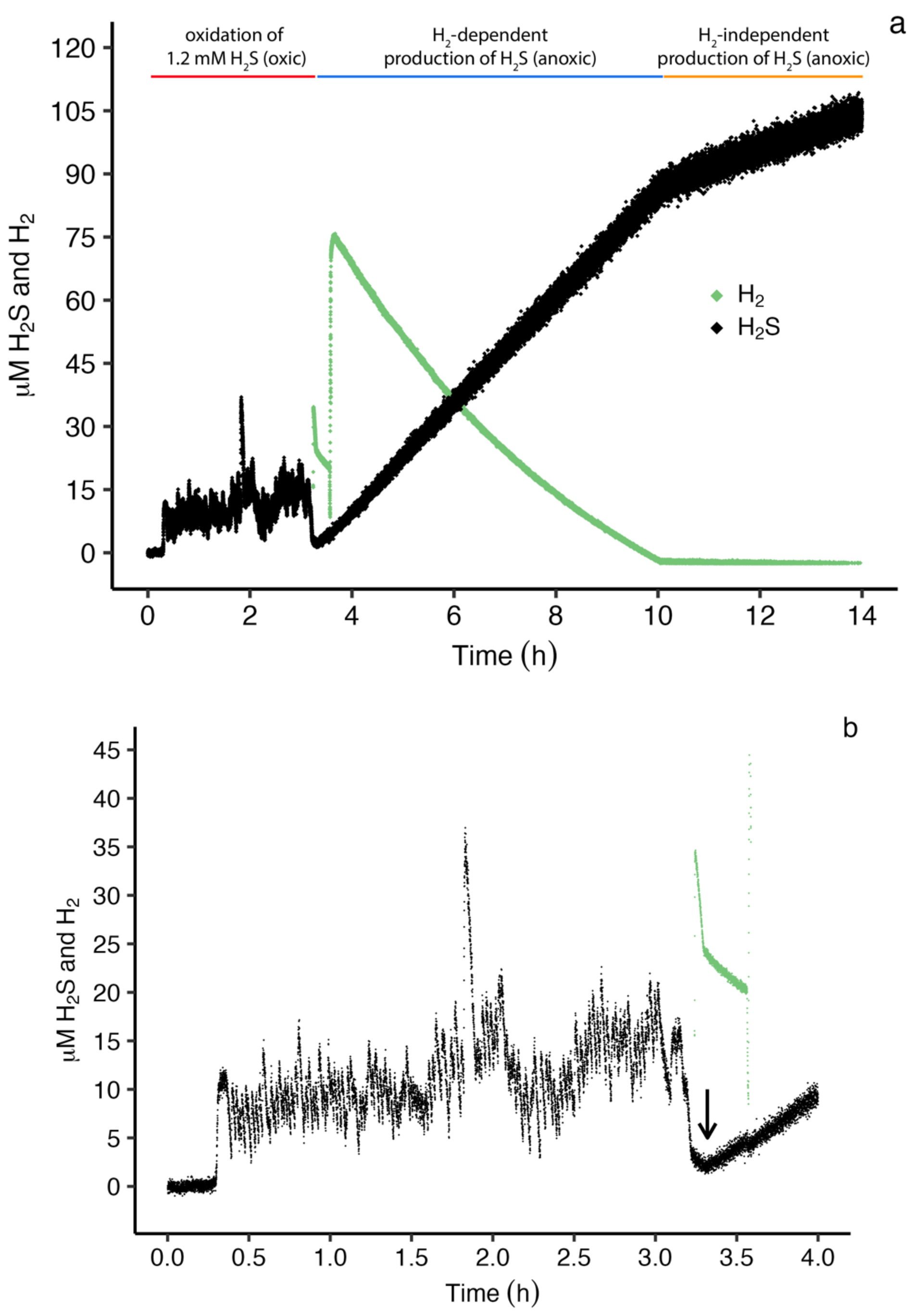

Supplementary Figure 7. Consumption and production of H_2S under oxic and anoxic conditions by sulfide-adapted *Methylacidiphilum fumariolicum* SolV cells. a In the first 3.2 hours, H_2S and O_2 were repeatedly spiked into the MIMS chamber, leading to aerobic H_2S consumption and the production of elemental sulfur and polysulfides. After 3.2 h, H_2 was twice injected, and the MIMS chamber rapidly became anoxic. From the moment of anoxia, H_2 consumption and concurrent H_2S production were observed. Upon H_2 depletion after 10 h, H_2S production decreased to lower rates. **b** Zoomed-in section of (**a**) in which aerobic H_2S oxidation took place. Arrow indicates the timepoint at which the MIMS chamber became anoxic, resulting in a lower H_2 consumption rate and shift from H_2S consumption to production. Source data are provided as a Source Data file.

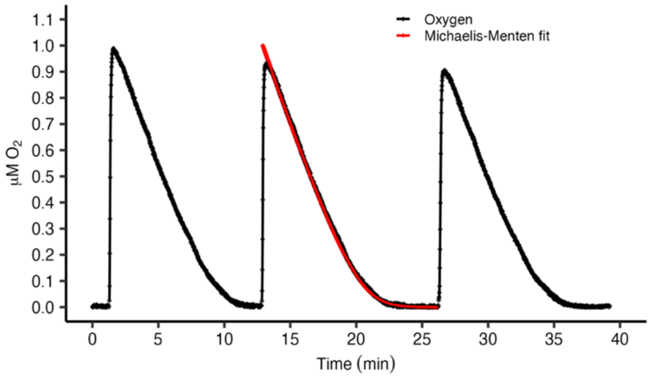

Supplementary Figure 8. Repeated respiration of H_2S by *M. fumariolicum* SolV cells in the presence of a starting concentration of 15 µM H_2S in the MIMS chamber. Oxygen was spiked after 1 min, 12.5 min and 26 min, and measured through a fiber-optic oxygen sensor spot. Red line indicates Michaelis-Menten curve fitting. Source data are provided as a Source Data file.

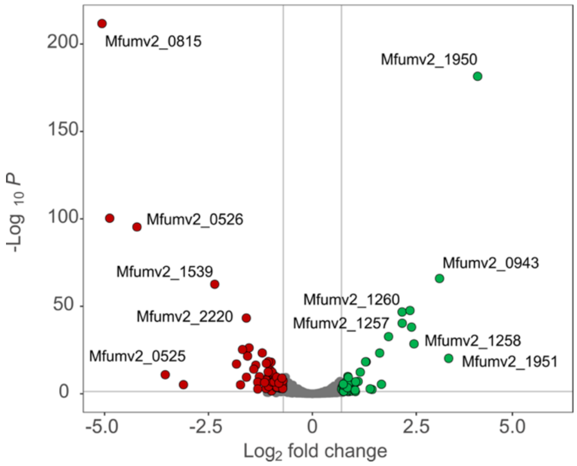

Supplementary Figure 9. **Volcano plot of gene regulation by *M. fumariolicum* SolV cells grown in the dual H_2S-CH_4 chemostat (sulfide-adapted cells) versus the CH_4 chemostat (non-adapted cells).** Green data points represent significantly upregulated genes; red datapoints represent significantly downregulated genes. A two-sided Wald test was performed by DEseq2 to calculate adjusted p-values.

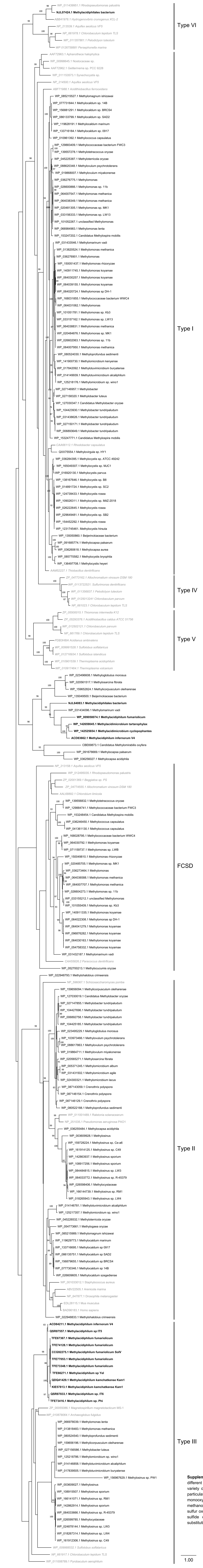

Supplementary Figure 10: Phylogenetic tree of amino acid sequences of different types of putative sulfide:quinone oxidoreductases (SQR) in a variety of organisms. A selection of microorganisms that also possess a particulate methane monooxygenase (pMMO) and/or soluble methane monooxygenase (sMMO) are indicated in black font. Verrucomicrobial methanotrophs are indicated in bold. Characterized non-methanotrophic sulfur oxidizing microbes are indicated in italics. FCSD: flavocytochrome *c* sulfide dehydrogenase. Scale bar indicates the average number of substitutions per site.
