# Supplementary material for: Simultaneous sulfide and methane oxidation by an extremophile
Source: Nat Commun. 2023 May 23;14:2974. doi: 10.1038/s41467-023-38699-9 (PMC10205796; doi:10.1038/s41467-023-38699-9)
Supplement: Supplementary file 3 — Description of Additional Supplementary Files [file 41467_2023_38699_MOESM3_ESM.pdf]

Description of Additional Supplementary Files:

Supplementary Dataset 1: Transcriptomic data obtained from *Methylacidiphilum fumariolicum* SolV cells grown in the dual CH_4-H_2S chemostat (sulfide-adapted cells) versus the CH_4 chemostat (non-adapted cells).
