# Supplementary material for: Simultaneous sulfide and methane oxidation by an extremophile
Source: Nat Commun. 2023 May 23;14:2974. doi: 10.1038/s41467-023-38699-9 (PMC10205796; doi:10.1038/s41467-023-38699-9)
Supplement: Supplementary file 5 — Reporting Summary [file 41467_2023_38699_MOESM5_ESM.pdf]

nature portfolio

Corresponding author(s): Prof. dr. H.J.M. Op den Camp

Last updated by author(s): May 8, 2023

Reporting Summary

Nature Portfolio wishes to improve the reproducibility of the work that we publish. This form provides structure for consistency and transparency in reporting. For further information on Nature Portfolio policies, see our Editorial Policies and the Editorial Policy Checklist.

Statistics

For all statistical analyses, confirm that the following items are present in the figure legend, table legend, main text, or Methods section.

n/a	Confirmed	
☐	☒	The exact sample size (*n*) for each experimental group/condition, given as a discrete number and unit of measurement
☐	☒	A statement on whether measurements were taken from distinct samples or whether the same sample was measured repeatedly
☐	☒	The statistical test(s) used AND whether they are one- or two-sided<br>*Only common tests should be described solely by name; describe more complex techniques in the Methods section.*
☒	☐	A description of all covariates tested
☒	☐	A description of any assumptions or corrections, such as tests of normality and adjustment for multiple comparisons
☐	☒	A full description of the statistical parameters including central tendency (e.g. means) or other basic estimates (e.g. regression coefficient) AND variation (e.g. standard deviation) or associated estimates of uncertainty (e.g. confidence intervals)
☐	☒	For null hypothesis testing, the test statistic (e.g. *F*, *t*, *r*) with confidence intervals, effect sizes, degrees of freedom and *P* value noted<br>*Give P values as exact values whenever suitable.*
☒	☐	For Bayesian analysis, information on the choice of priors and Markov chain Monte Carlo settings
☒	☐	For hierarchical and complex designs, identification of the appropriate level for tests and full reporting of outcomes
☒	☐	Estimates of effect sizes (e.g. Cohen's *d*, Pearson's *r*), indicating how they were calculated

Our web collection on statistics for biologists contains articles on many of the points above.

Software and code

Policy information about availability of computer code

Data collection	n/a
Data analysis	All software used is described in literature (BlastP, FastQC, Bowtie2, R 4.0.2, Rsubread, DEseq2, Muscle 3.8.1551, RAxML 8.2.10, MEGA7) and references are given in the text.

For manuscripts utilizing custom algorithms or software that are central to the research but not yet described in published literature, software must be made available to editors and reviewers. We strongly encourage code deposition in a community repository (e.g. GitHub). See the Nature Portfolio guidelines for submitting code & software for further information.

Data

Policy information about availability of data

All manuscripts must include a data availability statement. This statement should provide the following information, where applicable:

- Accession codes, unique identifiers, or web links for publicly available datasets
- A description of any restrictions on data availability
- For clinical datasets or third party data, please ensure that the statement adheres to our policy

The RNA sequencing data of this study have been deposited in the NCBI database under accession number PRJNA766544. The genome of Methylacidiphilum fumariolicum SolV has been deposited in the NCBI database under accession number ERS14853105. Source data are provided with this paper.

Human research participants

Policy information about studies involving human research participants and Sex and Gender in Research.

Reporting on sex and gender	n/a
Population characteristics	n/a
Recruitment	n/a
Ethics oversight	n/a

Note that full information on the approval of the study protocol must also be provided in the manuscript.

Field-specific reporting

Please select the one below that is the best fit for your research. If you are not sure, read the appropriate sections before making your selection.

☐ Life sciences ☐ Behavioural & social sciences ☒ Ecological, evolutionary & environmental sciences

For a reference copy of the document with all sections, see nature.com/documents/nr-reporting-summary-flat.pdf

Ecological, evolutionary & environmental sciences study design

All studies must disclose on these points even when the disclosure is negative.

Study description	Physiological experiments with Methylacidiphilum fumariolicum SolV
Research sample	A pure culture of the verrucomicrobial methanotroph Methylacidphilum fumariolicum SolV (Taxonomy ID: 1156937) was used for all experiments. The bacterium was originally isolated from a mudpot at the Solfatara volcano near Naple (Italy). The bacterium represents the dominant methanotroph in acidic volcanic ecosystems.
Sampling strategy	No sample size were calculated since we used a pure culture (as representive of methanotrophs in acidic volcanic ecosystems).All experiments involved batch and continuos cultivation of this bacterium. Cultivation methods are extensively described.
Data collection	Samples from the batch and chemostat cultures of Methylacidiphilum fumariolicum SolV were analyzed by Membrane inlet mass spectrometry, gas chromatography, respiration experiments, 13CO2 incorporation and TOC analysis by RA, AP, TvA, AI. The bioinformatic analyses were performed by RS, SP and TB.
Timing and spatial scale	n/a. For this study a pure culture was used. The results of our work can be extrapolated to the environment the bacterium was originally isloated from. As such timing and spatial scale are not relevant.
Data exclusions	No data were excluded.
Reproducibility	Each experiment was performed at least in duplicate and all replications of experiments were successful.
Randomization	n/a. For all experiments the same pure culture was used. As such randomization was not applicable.
Blinding	Binding is not relevant in this study once more since for all experiments a pure culture of Methylacidiphilum fumariolicum was used.

Did the study involve field work? ☐ Yes ☒ No

Reporting for specific materials, systems and methods

We require information from authors about some types of materials, experimental systems and methods used in many studies. Here, indicate whether each material, system or method listed is relevant to your study. If you are not sure if a list item applies to your research, read the appropriate section before selecting a response.

Materials & experimental systems

n/a	Involved in the study	
☒	☐	Antibodies
☒	☐	Eukaryotic cell lines
☒	☐	Palaeontology and archaeology
☒	☐	Animals and other organisms
☒	☐	Clinical data
☐	☒	Dual use research of concern

Methods

n/a	Involved in the study	
☒	☐	ChIP-seq
☒	☐	Flow cytometry
☒	☐	MRI-based neuroimaging

Dual use research of concern

Policy information about dual use research of concern

Hazards

Could the accidental, deliberate or reckless misuse of agents or technologies generated in the work, or the application of information presented in the manuscript, pose a threat to:

No	Yes	
☒	☐	Public health
☒	☐	National security
☒	☐	Crops and/or livestock
☒	☐	Ecosystems
☒	☐	Any other significant area

Experiments of concern

Does the work involve any of these experiments of concern:

No	Yes	
☒	☐	Demonstrate how to render a vaccine ineffective
☒	☐	Confer resistance to therapeutically useful antibiotics or antiviral agents
☒	☐	Enhance the virulence of a pathogen or render a nonpathogen virulent
☒	☐	Increase transmissibility of a pathogen
☒	☐	Alter the host range of a pathogen
☒	☐	Enable evasion of diagnostic/detection modalities
☒	☐	Enable the weaponization of a biological agent or toxin
☒	☐	Any other potentially harmful combination of experiments and agents
